# Developed-developing country partnerships: benefits from South to North?

**DOI:** 10.1186/1753-6561-5-S6-O23

**Published:** 2011-06-29

**Authors:** SB Syed, V Dadwal, D Pittet

**Affiliations:** 1 WHO, Geneva, Switzerland; 2 University of Geneva Hospitals, Geneva, Switzerland

## Introduction / objectives

Developed-developing country partnerships can act as a platform for realizing mutual benefits and contribute to global health. Benefits accrued by developed countries from partnering with developing countries are often unknown.

## Methods

A literature review was conducted using PubMed database, grey literature, media scanning and cited references. A standardized approach was utilized to extract key points from each article to understand benefits accrued by developed countries. Benefits were categorized and interpreted using a hybrid categorization framework combining the Partnership Evaluation Tool (PET) and the WHO health systems framework. Findings were further examined for applicability to infection prevention and control practice.

## Results

The review showcased instances of direct benefits accruing to individuals or organisations involved in partnerships. More importantly, the review demonstrated possibilities for system-wide benefits to developed countries in each of the six health system building blocks. Whether it be service delivery; health workforce; health information; medical products, vaccines and technologies; financing; or leadership— opportunities for networking, learning and action constitute the foundation of strong partnerships between developed and developing countries. These findings have particular relevance to knowledge flow from developing to developed countries on infection prevention and control.

## Conclusion

Learning to value all forms of knowledge is essential if we are to redesign conventional practices. In this regard, developing countries are proving their ability to lead change. This realization can transform current paradigms on the nature of learning and global knowledge transfer. The global infection prevention and control community can lead this paradigm shift to enhance global health.

## Disclosure of interest

None declared.

